# Digital microfluidics-engaged automated enzymatic degradation and synthesis of oligosaccharides

**DOI:** 10.3389/fbioe.2023.1201300

**Published:** 2023-06-21

**Authors:** Yunze Sun, Yiran Wu, Dachuan Ma, Jian-Jun Li, Xianming Liu, Yuanjiang You, Jun Lu, Zhen Liu, Xin Cheng, Yuguang Du

**Affiliations:** ^1^ School of Chemical Engineering, University of Chinese Academy of Sciences, Beijing, China; ^2^ State Key Laboratory of Biochemical Engineering, Institute of Process Engineering, Chinese Academy of Sciences, Beijing, China; ^3^ Department of Biotechnology, Dalian Institute of Chemical Physics, Chinese Academy of Sciences, Dalian, Liaoning, China; ^4^ Institute of Physics, Chinese Academy of Sciences, Beijing, China; ^5^ Department of Materials Science and Engineering, Southern University of Science and Technology, Shenzhen, China

**Keywords:** digital microfluidics (DMF), oligosaccharides synthesis, oligosaccharides degradation, exoglycosidase digestion, glycosyltransferase (GT), enzymatic module

## Abstract

Glycans are an important group of natural biopolymers, which not only play the role of a major biological energy resource but also as signaling molecules. As a result, structural characterization or sequencing of glycans, as well as targeted synthesis of glycans, is of great interest for understanding their structure–function relationship. However, this generally involves tedious manual operations and high reagent consumptions, which are the main technical bottlenecks retarding the advances of both automatic glycan sequencing and synthesis. Until now, automated enzymatic glycan sequencers or synthesizers are still not available on the market. In this study, to promote the development of automation in glycan sequencing or synthesis, first, programmed degradation and synthesis of glycans catalyzed by enzymes were successfully conducted on a digital microfluidic (DMF) device by using microdroplets as microreactors. In order to develop automatic glycan synthesizers and sequencers, a strategy integrating enzymatic oligosaccharide degradation or synthesis and magnetic manipulation to realize the separation and purification process after enzymatic reactions was designed and performed on DMF. An automatic process for enzymatic degradation of tetra-*N*-acetyl chitotetraose was achieved. Furthermore, the two-step enzymatic synthesis of lacto-*N*-tetraose was successfully and efficiently completed on the DMF platform. This work demonstrated here would open the door to further develop automatic enzymatic glycan synthesizers or sequencers based on DMF.

## 1 Introduction

Glycans, nucleic acids, and proteins are three major classes of natural biopolymers (Wen et al., 2018). In addition to their traditionally recognized roles as energy sources for living organisms, it is now well known that glycans play important signaling roles in a variety of physiological and pathological processes, including cell growth and proliferation (Varki, 2017), immune responses (Crocker et al., 2007), angiogenesis and tumor cell metastasis (Lou et al., 2014), protein folding and degradation (Parodi, 2000), cell–cell communications (Cannon and Helenius, 1999), and cell–pathogen interactions (Kato and Ishiwa, 2015). Identifying links between glycan structures and functions (Dennis et al., 2009), monitoring glycosylation in disease diagnosis and prognosis (Peracaula et al., 2008), and elucidating molecular mechanisms of glycans with structural differences involved in pathogenesis (Freeze and Aebi, 2005) are highlighted in current glycan-related studies. Therefore, developing precise, robust, and sensitive methodologies for the analysis and synthesis of glycans with definite structures is critical.

However, the extremely high diversity of isomerization makes structural elucidation of glycans a challenging job. The most frequently used methods for the structural analysis of glycans include capillary electrophoresis (CE) (Guttman, 1997), nuclear magnetic resonance spectroscopy (NMR) (Wyss et al., 1995), mass spectrometry (MS) (Morelle and Michalski, 2007), and liquid chromatography (LC) (Royle et al., 2008), which are often combined when being applied (Houel et al., 2014). In order to identify glycosidic linkages, an exoglycosidase-based digestion technique by highly specific non-reducing end exoglycosidase is routinely used for glycan sequencing coupled with the aforementioned methods (Tzur et al., 2008). However, this approach often involves tedious and time-consuming manual processes (Szigeti and Guttman, 2017).

The availability of pure glycans is a prerequisite for understanding the molecular details of their functions (Rillahan and Paulson, 2011) and producing homogeneous glycoconjugates (Kosik et al., 2010). The chemical synthesis of oligosaccharides generally consists of a glycosyl donor, a glycosyl acceptor, and an activator (Wen et al., 2018). Numerous chemical strategies have been explored for the assembly of complex glycans, such as Schmidt’s imidate (Hummel and Schmidt, 1997), Nicolaou’s two-stage activation (Nicolaou and Mitchell, 2001), Danishefsky’s glycal assembly (Danishefsky and Bilodeau, 1996), Kahne’s sulfoxide glycosylation (Yan and Kahne, 1996), Boons’ polymer-supported solution-phase synthesis (Zhu and Boons, 2000), Ye and Huang’s preactivation glycosylation (Miermont et al., 2007), and Wong’s reactivity-based one-pot synthesis (Koeller and Wong, 2000). Compared with the chemical synthesis of glycans, enzymatic synthesis has attracted more attention due to high stereoselectivity, high regioselectivity, non-protection, mild reaction conditions, and environmental friendliness (Chen, 2015). In recent years, one-pot multienzyme (OPME) systems for oligosaccharide synthesis have been well established (Yu and Chen, 2016), which allow the use of designated enzymatic modules for extending specific monosaccharides with specific configurations and linkages on the glycan (Ye et al., 2019).

Tedious procedures and high labor costs are common technical limitations to rapid advances in both glycan sequencing and synthesis. Thus, automatic strategies have been developed for glycan synthesis. The first automated glycan synthesizer was set up by modifying a peptide synthesizer based on a solid-phase synthetic method (Plante et al., 2001). Later, the enzymatic synthesis of oligosaccharides was achieved in an automated manner using a commercially available peptide synthesizer (Zhang et al., 2018). More recently, based on an ISYNTH AI SWING workstation (a liquid handling system), an automated platform for enzyme-mediated oligosaccharide synthesis was built (Li et al., 2019). By contrast, automated glycan sequencers have been not reported yet, even though some explorations have been made for automatic glycan sequencing. For instance, a novel automated approach for *N*-glycan sequencing of biopharmaceuticals by combining capillary electrophoresis and exoglycosidases has been reported (Szigeti and Guttman, 2017). Miniaturized platforms for implementing enzyme-promoted synthesis and degradation of oligosaccharides with high automation and reliability are strongly needed for processing a small number of samples. Chemists have long been interested in miniaturizing chemical reactions to take advantage of favorable scaling of diffusion and heat exchange (Davies, 2019; Hardwick and Ahmed, 2020).

Digital microfluidics (DMF) is a novel microscale liquid-handling platform on which picoliter- and microliter-sized droplets are manipulated on arrays of electrodes coated with a hydrophobic insulator (Samiei et al., 2016). On a DMF chip, droplets can be manipulated to move, dispense, mix, split, and merge with ease of automation. DMF is being extensively used to miniaturize a wide range of biochemical applications, such as PCR (Sanders et al., 2011), single cell analysis and immunoassay (He et al., 2015), and chemical synthesis of peptides (Jebrail et al., 2012; Xing et al., 2021), with the advantages of much less amount of sample consumptions, faster heat and mass transfer and reaction rates, and higher integration capacity. To our knowledge, there has been no precedence for enzymatic degradation and synthesis of oligosaccharides reported on the DMF platform. In this report, the viability of using the DMF platform for enzymatic degradation and synthesis of oligosaccharides was investigated.

## 2 Materials and methods

### 2.1 Materials and reagents

Unless specified, all chemicals were of analytical grade and purchased from Sigma (St. Louis, Missouri, United States), Aladdin (Shanghai, China), or Beijing Solarbio Science and Technology Co. Ltd (Beijing, China). Tetra-*N*-acetyl chitotetraose was purchased from Mega (Bray, Ireland). Chitosan oligosaccharide RX14 was obtained from the company China RongXin Biotechnology Co., Ltd. (Suzhou, China). 7-Aminonaphthalene-1,3-disulfonic acid (Tag1) was purchased from TCI Development Co., Ltd. (Shanghai, China). Tag2 (7-(2-(2-(*N*-methyl-aminooxy)ethoxy)ethoxy)naphthalene-1,3-disulfonic acid) was synthesized by Shanghai Nafu Biotechnology Co. Ltd. according to the published procedure (Li et al., 2019) (Shanghai, China).

### 2.2 Establishment of the DMF device

The DMF platform was set up according to the published procedure (Supplementary Figure S1) (Li et al., 2022).

On the two-plate DMF device used for enzymatic reactions, 80 actuation electrodes were fabricated on the bottom plate, which were covered with 8 μm thick SU-8 as the dielectric layer and 50 nm thick Teflon as the hydrophobic layer to prevent damage to the electrodes, while the top plate was also covered with the same hydrophobic layer. Four semicircular notches and four through holes were fabricated on the top plate with a laser scriber for droplet loading and collecting with a pipette. The device was assembled by joining a top and bottom plate with a spacer formed by three layers of double-sided tape with a thickness of approximately 0.15 mm. Accordingly, the volume of one droplet on one electrode was around 2–2.5 μL.

### 2.3 Production and purification of enzymes

*Streptomyces alfalfae β*-1,4-*N*-acetylglucosaminidase (GlcNAcase) (Lv et al., 2019), *Trichoderma reesei β*-1,4-glucosaminidase (GlcNase) (Ike et al., 2006), *Bifidobacterium longum N*-acetylhexosamine-1-kinase (NahK) (Li et al., 2011), *Pasteurella multocida N*-acetylglucosamine uridylyltransferase (GlmU) (Chen et al., 2011), *Helicobacter pylori β*-1,3-*N*-acetylglucosaminyltransferase (LgtA) (Li et al., 2016), *Escherichia coli* K-12 pyrophosphatase (PpA) (Li et al., 2013), *Escherichia coli* K-12 galactokinase (GalK) (Chen et al., 2002), *Bifidobacterium longum* UDP-sugar pyrophosphorylase (USP) (Muthana et al., 2012), and *Escherichia coli* O55:H7 *β*-1,3-galactosyltransferase (WbgO) (Liu et al., 2009) were expressed and purified as reported in the literature.

In general, all enzymes were overexpressed in *E. coli* BL21(DE3) and induced by isopropyl 1-thio-D-galactopyranoside (IPTG) according to the aforementioned published procedures. The cells were collected by centrifugation and suspended in the equilibration buffer (50 mM NaH_2_PO_4_, 300 mM NaCl, and 10 mM imidazole, pH 8.0). Cells were disrupted by sonication and centrifuged at 15,000 g for 20 min at 4°C to remove insoluble cell debris. The supernatants were applied to a Ni^2+^-NTA agarose column and washed with equilibration buffer and elution buffer (50 mM NaH_2_PO_4_, 300 mM NaCl, and 50/200 mM imidazole, pH 8.0) in turn to remove protein impurities. The fractions showing enzyme activities were pooled and concentrated. SDS-PAGE was carried out on a 12% separation gel. Protein concentration was measured by using the Bradford method with bovine serum albumin as the standard.

### 2.4 Droplet operation on the DMF device

The droplets were actuated with a high-voltage alternative wave generator (QFC 2020D, Dalian Quantum Fluid Control Technology, China) in a sinusoid waveform (300 V RMS with a frequency of 1 kHz). The actuation of droplets started from loading deionized water to test the actuation versatility of the DMF device. The reagents used on the DMF device included saccharides, salts, enzymes, and magnetic beads. For saccharides, 100 mM glucose, 100 mM galactose, 100 mM lactose, and 10 mg/mL RX14 were tested, respectively. For salts, 50 mM NaOAc buffer (pH = 5.5), 1 M Tris-HCl buffer (pH = 8.0), 60 mM NH_4_HCO_3_, and 100 mM MgCl_2_ were tested, respectively. For enzymes, each enzyme mentioned previously was tested. Before loading, Pluronic F127 surfactant was added at a concentration of 0.05% (v/v).

### 2.5 Enzymatic degradation of oligosaccharides on the DMF device

Tetra-*N*-acetyl chitotetraose (A4) at a concentration of 5 mg/mL was loaded in one droplet, and GlcNAcase at a concentration of 0.1 mg/mL in NaOAc buffer (50 mM, pH = 5.5) was loaded in another one.

As for another reaction, chitosan oligosaccharide RX14 at a concentration of 5 mg/mL was loaded in one droplet, and GlcNAcase and GlcNase at a concentration of 0.1 mg/mL in NaOAc buffer (50 mM, pH = 5.5) was loaded in another one.

On the DMF platform, the two droplets were mixed evenly and left for 1 h for enzymatic degradation. After the reaction, the droplet containing the degradation product was taken out through a hole on the top plate and characterized by MS.

### 2.6 Enzymatic synthesis of oligosaccharides on the DMF device

Substrates and enzymatic modules were loaded in two droplets. One droplet contained 3 mM lactose, 3 mM *N*-acetylglucosamine, 3.6 mM ATP, and 3.6 mM UTP, while another one contained enzymatic module 1 (Supplementary Table S1), 10 mM MgCl_2_, and 50 mM NH_4_HCO_3_.

Like the process for oligosaccharide degradation, the two droplets were mixed evenly and left for 1 h for enzymatic synthesis. After the reaction, the droplet containing the synthesized product was taken out through a hole on the top plate and characterized by mass spectrometry.

### 2.7 Synthesis of oligosaccharide modified with Tags

Tag1 (0.03 mmol) and tetra-*N*-acetyl chitotetraose (A4, 0.0024 mmol) were dissolved in 1 mL 15% CH_3_COOH solution. Next, sodium cyanoborohydride (NaBH_3_CN, 0.03 mmol) was added. The reaction mixture was incubated at 50°C for 15 h (Chiesa and Oneill, 1994). The reaction mixture was purified on a Bio-Gel P-2 column from Bio-Rad (Hercules, California, United States). The column was eluted with water. The fractions containing the product were combined and lyophilized. The product, A4-Tag1, was confirmed by MS (Supplementary Figure S2).

Tag2 (0.008 mmol) was dissolved in NaOAc buffer (100 mM, pH 4.2, 100 µL). Next, lactose (Lac, 0.08 mmol) was added. The reaction mixture was incubated at 37°C for 24 h (Li et al., 2019). The reaction mixture was purified on a Bio-Gel P-2 column. The column was eluted with water. The fractions containing the product were combined and lyophilized. The product, Lac-Tag2, was confirmed by MS (Supplementary Figure S3).

### 2.8 Automatic enzymatic degradation of oligosaccharides on the DMF device

Tetra-*N*-acetyl chitotetraose modified with Tag1 (A4-Tag1) at a concentration of 1 mg/mL was loaded in one droplet, while GlcNAcase at a concentration of 0.02 mg/mL in NaOAc buffer (50 mM, pH = 5.5) was loaded in another droplet.

On the DMF platform, the two droplets were mixed evenly and left for 1 h for enzymatic degradation. Then reaction mixture was mixed with DEAE (diethylaminoethyl) magnetic beads in one droplet, which were suspended in the droplet to capture products modified with Tag1. The DEAE magnetic beads and supernatant were separated by a magnet, and the supernatant containing the degradation products was taken out through a hole on the top plate for MS analysis. Subsequently, DEAE magnetic beads were washed in turn with water and 60 mM NH_4_HCO_3_. Finally, the captured products on DEAE magnetic beads were released by washing with 0.3 M NH_4_HCO_3_, and the eluent obtained was transferred and characterized by MS.

### 2.9 Automatic enzymatic synthesis of oligosaccharides on the DMF device

Lactose modified with Tag2 (Lac-Tag2) at a concentration of 3 mM was loaded in the first droplet. Enzymatic module 1, 10 mM MgCl_2_, 50 mM NH_4_HCO_3_, 3 mM *N*-acetylglucosamine, 3.6 mM ATP, and 3.6 mM UTP were loaded in the second droplet. Enzymatic module 2, 10 mM MgCl_2_, 50 mM NH_4_HCO_3_, 3 mM galactose, 3.6 mM ATP, and 3.6 mM UTP were loaded in the third droplet. The components of the enzymatic modules are shown in Supplementary Table S2.

On the DMF platform, the first two droplets were mixed evenly and left for 1 h for enzymatic synthesis. Then the reaction mixture was mixed with DEAE magnetic beads in one droplet. The DEAE magnetic beads were suspended in the droplet to capture the products modified with Tag2. The DEAE magnetic beads and supernatants were separated by a magnet, and the supernatant, including enzymes, excess (sugar) nucleotides, MgCl_2_, and NH_4_HCO_3_, was taken out and discarded. Subsequently, DEAE magnetic beads were washed in turn with water and 60 mM NH_4_HCO_3_. Next, the captured products with Tag2 on DEAE magnetic beads were released by washing with 0.3 M NH_4_HCO_3_, and the eluent obtained was mixed evenly with the third droplet and left for 1 h for the next enzymatic synthesis reaction. Like the first cycle, DEAE magnetic beads were used for capturing the synthesized products. Finally, the eluent washed by 0.3 M NH_4_HCO_3_ was transferred and characterized by MS.

### 2.10 Removal of Tag2 after enzymatic synthesis on the DMF device

The eluent from 2.9 containing lacto-*N*-tetraose modified with Tag2 (LNT-Tag2) was mixed with a droplet (2 μL) of trifluoroacetic acid (0.25%, v/v) on the DMF platform. The reaction mixture was agitated at room temperature for 2 h (Prudden et al., 2014). After the reaction, the product was mixed with a droplet of 50 mM NH_4_HCO_3_ to adjust the pH. Then, DEAE magnetic beads were used for capturing Tag2 removed from LNT-Tag2, and the supernatant including lacto-*N*-tetraose (LNT) was taken out and characterized by MS.

### 2.11 MS analysis of the products in droplets

Analysis of the products was performed on the UltiMate-3000-ISQ-EM (Thermo Fisher Scientific). The droplets were diluted to 100 μL before analysis. Samples were loaded directly without columns. The mobile phase was water, and the flow rate was 0.1 mL/min.

## 3 Results and discussion

### 3.1 Droplet operation on the DMF chip

The two-plate DMF device was established according to a published procedure (Li et al., 2022).

A droplet on a digital microfluidic chip was actuated under the electrowetting mechanism (Wheeler, 2008), by which the wettability of its static hydrophobic surface can be tuned to hydrophilic under an applied electric field, providing additional charges into the triple contact line of the droplet, the chip surface, and the surrounding oil. Under the two-plate format, as in our DMF chip, when the adjacent electrode of a droplet is charged, the electrocapillary force on the triple line breaks the horizontal force balance and pulls the droplet onto the activated electrode. This is the basic working mechanism of droplet actuation. When two droplets are transported onto the same electrode, they are fused into one bigger droplet. Therefore, when the two droplets carrying two reactants, respectively, are merged, a reaction takes place in the merged droplet.

Initially, droplets containing different substances, including saccharides, enzymes, and salts, involved in enzymatic synthesis and degradation of oligosaccharides were manipulated on the DMF platform to test the feasibility of our methodology. Droplets containing salts or saccharides can be actuated smoothly in both air and silicone oil (cSt 1.5), while those containing enzymes can only be actuated in silicone oil after being doped with 0.05% Pluronic F127 (Luk et al., 2008). In addition, evaporation of droplets on the DMF chip is an issue. In the air, a droplet with a volume of 2 μL can only be kept for around 1–2 h at room temperature. In order to avoid evaporation of droplets, the droplets can be immersed in silicone oil so that they can be kept for at least 10 h at room temperature. In order to be compatible with the DMF platform, the droplets of the enzymatic reactions were conducted in the presence of 0.05% Pluronic F127 in silicone oil in the following experiments unless indicated otherwise.

### 3.2 Enzymatic degradation and synthesis of oligosaccharides on the DMF chip

**FIGURE 1:**
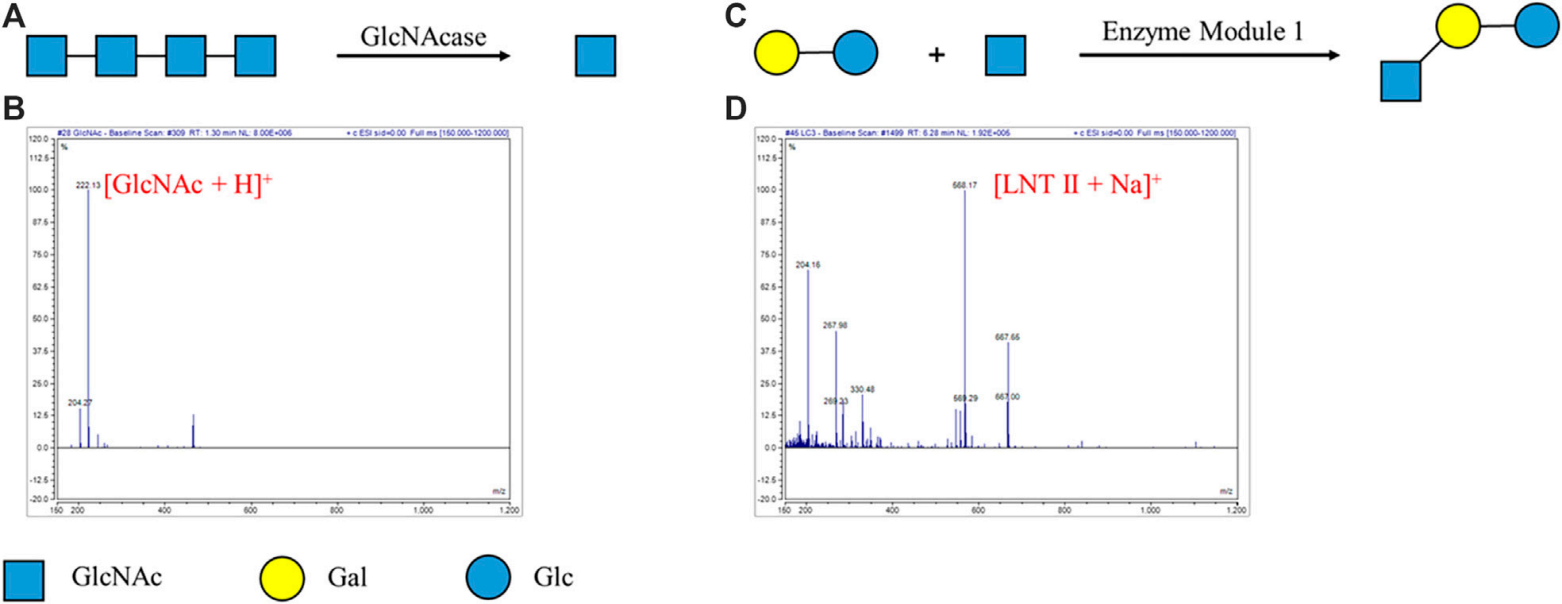
Enzymatic reaction for degradation and synthesis of oligosaccharides on the DMF device. **(A)** GlcNAcase-catalyzed degradation of tetra-*N*-acetyl chitotetraose (A4). **(B)** MS characterization of GlcNAcase-catalyzed degradation of tetra-*N*-acetyl chitotetraose (A4). ESI-MS *m/z* calculated for C_8_H_16_NO_6_ [M + H]^+^ 222.22 and found to be 222.13. The peak corresponding to A4 (calculated as [M + H]^+^ 831.80) was not detected. **(C)** Enzymatic module-catalyzed synthesis of lacto-N-triose II (LNT II). **(D)** MS characterization of enzymatic module-catalyzed synthesis of lacto-N-triose II (LNT II). ESI-MS *m/z* calculated for C_20_H_35_NO_16_Na [M + Na]^+^ 568.48 and found to be 568.17. The peak corresponding to lactose (calculated as [M + Na]^+^ 365.29) was not detected.

We tried degradation of oligosaccharides by specific non-reducing end exoglycosidases on the DMF platform. Specific non-reducing end GlcNAcase (β-1,4-*N*-acetylglucosaminidase) (EC 3.2.1.52) was used to hydrolyze tetra-*N*-acetyl chitotetraose (A4) into *N*-acetylglucosamine (GlcNAc) (Figure 1A). A4 was loaded in a droplet, and GlcNAcase and a buffer were carried in another droplet. The two droplets were mixed evenly and incubated for enzymatic degradation of A4. After 1 h, the droplet containing the degradation product was taken out through a hole on the top plate and characterized by mass spectrometry (Supplementary Video S1). According to MS characterization of GlcNAcase-catalyzed degradation of tetra-*N*-acetyl chitotetraose (A4), ESI-MS *m/z* was calculated for the degradation product GlcNAc (C_8_H_16_NO_6_) as [M + H]^+^ 222.22 and found to be 222.13. The peak corresponding to A4 (calculated as [M + H]^+^ 831.80) was not detected based on MS. MS analysis indicated that the degradation product GlcNAc was detected and enzymatic activities were not affected by the electric field (Figure 1B). Similarly, specific non-reducing end GlcNase (β-1,4-glucosaminidase) (EC 3.2.1.165) was used to degrade chitosan oligosaccharide RX14, and the product glucosamine (GlcN) was observed (Supplementary Figure S4). These experiments demonstrated that enzymatic degradation of oligosaccharides in one droplet was successfully achieved on our DMF platform.

Encouraged by these results, enzymatic module-catalyzed synthesis of oligosaccharide lacto-*N*-triose II (LNT II) was also attempted (Figure 1C). The substrates, including lactose, GlcNAc, ATP, and UTP, were loaded in a droplet, while the enzymatic module 1 (including NahK, GlmU, LgtA, PpA), MgCl_2_, and buffer were put in another one. The ratio of different enzymes in the enzymatic modules was optimized for efficient enzymatic synthesis. The two droplets were mixed and incubated for 1 h and characterized by mass spectrometry. According to MS characterization of enzymatic module-catalyzed synthesis of lacto-N-triose II (LNT II), ESI-MS *m/z* was calculated for LNT II (C_20_H_35_NO_16_Na) as [M + Na]^+^ 568.48 and found to be 568.17. The peak corresponding to lactose (calculated as [M + Na]^+^ 365.29) was not detected based on MS (Figure 1D). MS analysis indicated that nearly all substrates were converted into the product LNT II.

### 3.3 Automated enzymatic degradation and synthesis of oligosaccharides on the DMF chip

**FIGURE 2:**
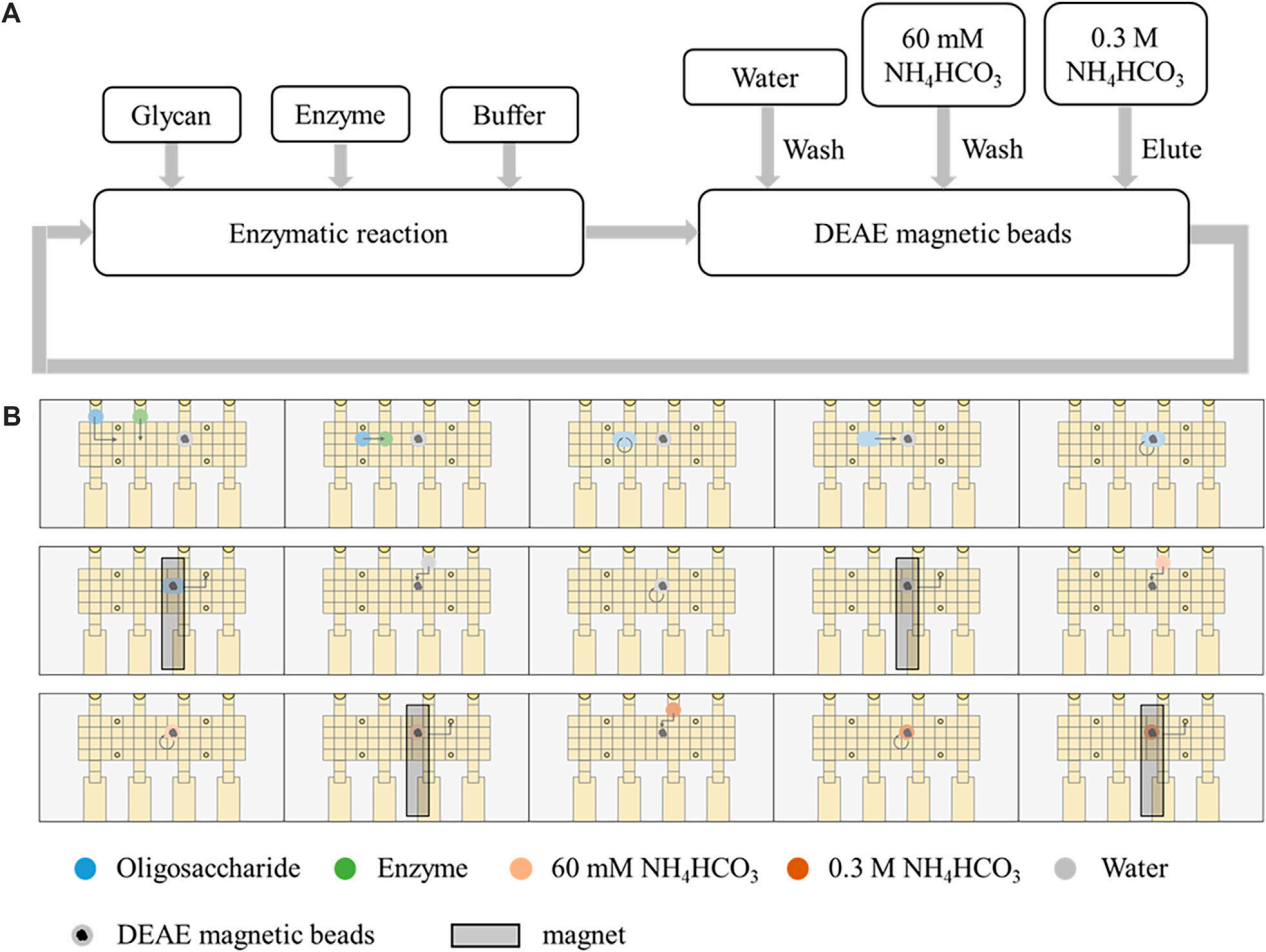
Automatic strategy coupling enzymatic reactions and magnetic manipulation on the DMF device. **(A)** Schematic processes of enzymatic reactions and separation by DEAE magnetic beads. **(B)** Schematic of the processes on the DMF device.

**FIGURE 3:**
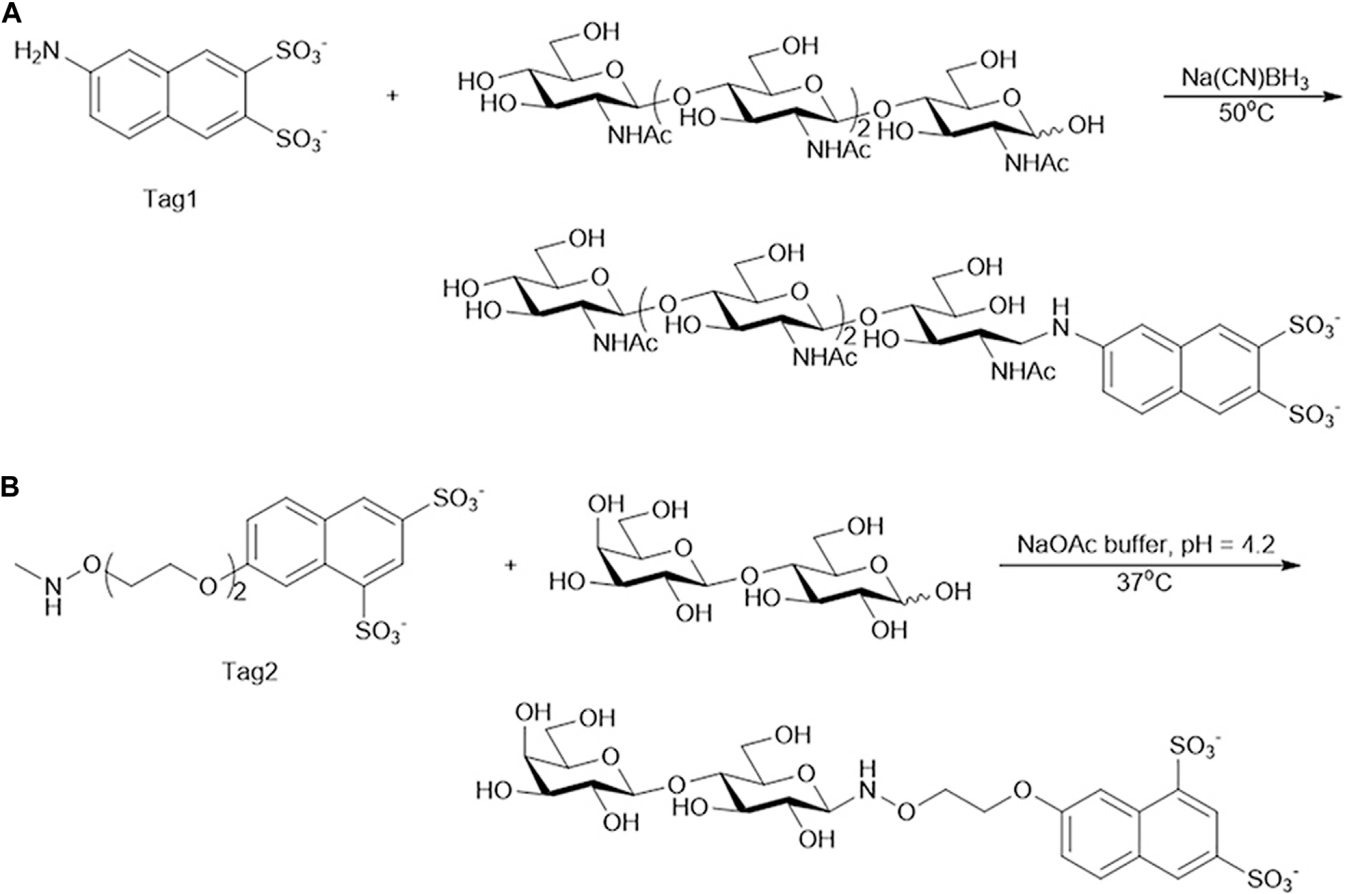
Synthesis of oligosaccharide modified with Tag. **(A)** Tetra-*N*-acetyl chitotetraose (A4) modified with Tag1. **(B)** Lactose modified with Tag2.

In order to develop automatic glycan synthesizers and sequencers, a strategy integrating enzymatic oligosaccharide degradation or synthesis and magnetic manipulation to realize the separation and heterogeneous purification process after enzymatic reactions on the DMF platform was designed (Figure 2). The sulfonate tags bear two sulfonate groups, which can be captured onto anion exchange resin and released by 0.3 M NH_4_HCO_3_. The sulfonated tags can be efficiently attached to the reducing ends of the starting saccharides (Figure 3), which have been demonstrated not to interfere with enzymatic reactions (Chiesa and Oneill, 1994; Li et al., 2019).

**FIGURE 4:**
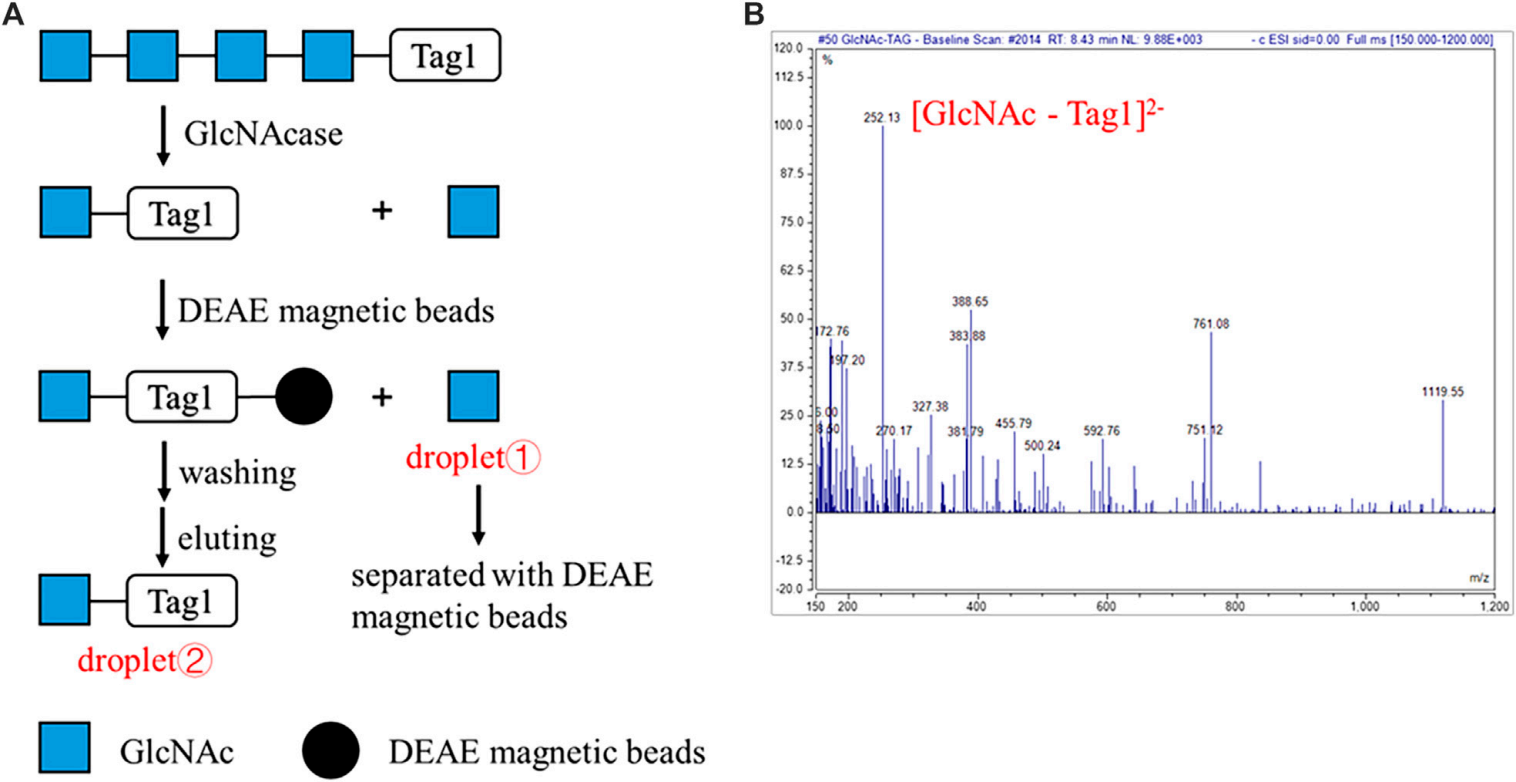
Automatic strategy of enzymatic reaction for oligosaccharides degradation on the DMF device. **(A)** Schematic processes of the automatic strategy of GlcNAcase-catalyzed degradation of tetra-*N*-acetyl chitotetraose (A4). **(B)** MS characterization of the product GlcNAc-Tag1. ESI-MS *m/z* calculated for C_18_H_20_N_8_O_11_S_2_ [M - 2H]^2−^ 252.25 and found to be 252.13. The peak corresponding to A4-Tag1 (calculated for C_42_H_59_N_5_O_26_S_2_ as [M - 2H]^2−^ 557.03) was not detected.

To verify this automatic process, GlcNAcase was still used to degrade A4 modified at the reducing end with the sulfonate Tag1 (A4-Tag1) on the DMF platform (Figure 4A). The process on the DMF platform was shown in Supplementary Video S2. First, the droplet containing A4-Tag1 and another one containing GlcNAcase and buffer were mixed and incubated for 1 h. Then, DEAE (diethylaminoethyl) magnetic beads were suspended in the droplet to capture the products modified with Tag1. The DEAE magnetic beads and supernatants were separated by a magnet, and the supernatant droplet ① containing GlcNAc cleaved off from the non-reducing end of A4-Tag1 by GlcNAcase was taken out through a hole on the top plate for MS analysis. Subsequently, DEAE magnetic beads were washed in turn with water and 60 mM NH_4_HCO_3_. Finally, the captured products with Tag1 on DEAE magnetic beads were released by washing with a small volume of 0.3 M NH_4_HCO_3_, and the eluted droplet ② obtained was transferred and characterized by MS. According to MS results (Figure 4B; Supplementary Figure S5), GlcNAc (calculated as [M + H]^+^ 222.22 and found to be 222.05) was found in the droplet ① and GlcNAc-Tag1 (calculated for C_18_H_20_N_8_O_11_S_2_ as [M - 2H]^2−^ 252.25 and found to be 252.13) was also detected in droplet ②. In addition, the peak corresponding to A4-Tag1 (calculated for C_42_H_59_N_5_O_26_S_2_ as [M - 2H]^2−^ 557.03) was not detected based on MS in droplet ②. These MS results proved that A4-Tag1 in a single droplet was broken down into GlcNAc and GlcNAc-Tag1, and the degradation products were completely separated by DEAE magnetic beads through an automatic process on the DMF platform.

**FIGURE 5:**
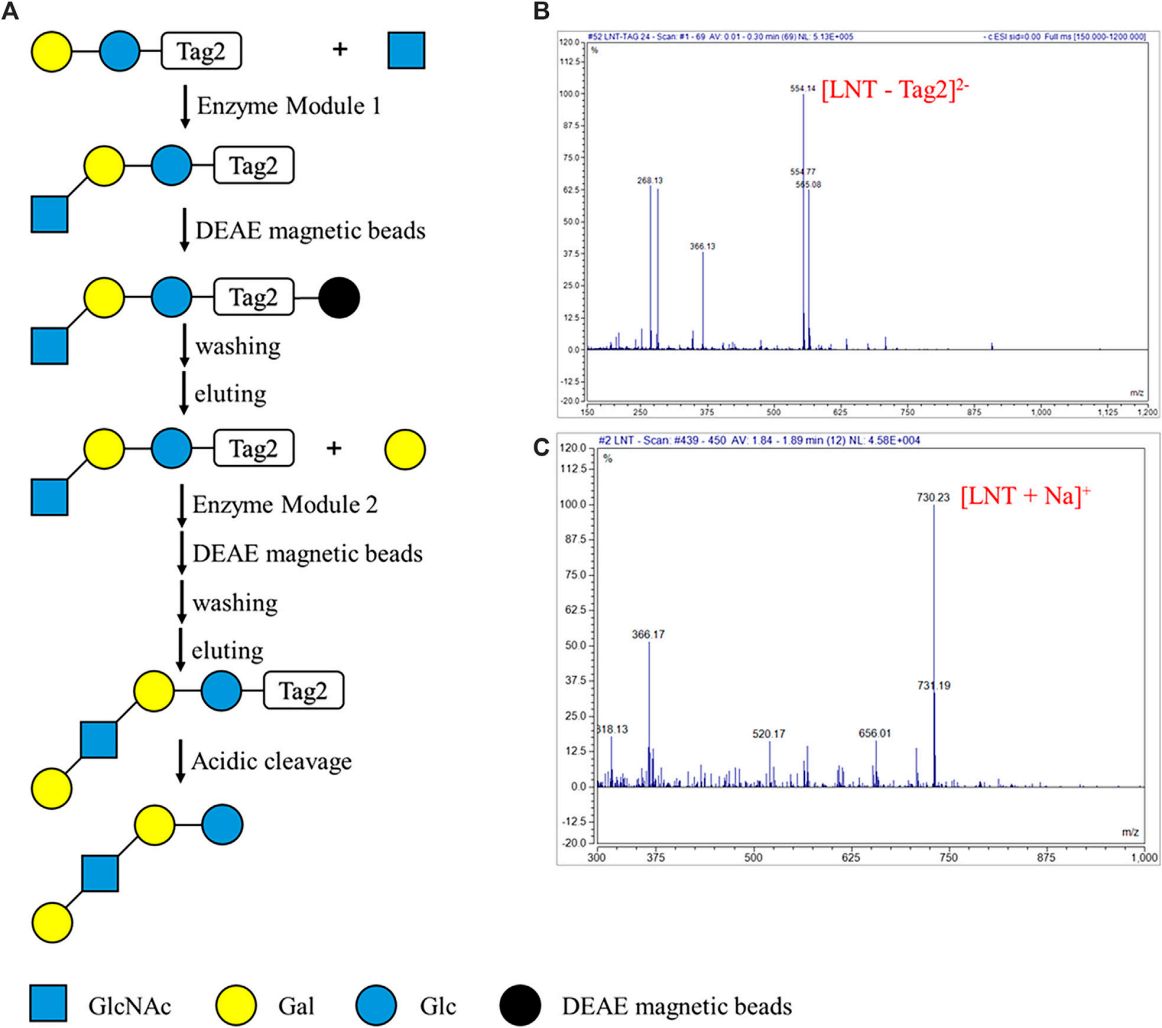
Automatic strategy of enzymatic reaction for oligosaccharides synthesis on the DMF device. **(A)** Schematic processes of the automatic strategy of enzymatic module-catalyzed synthesis of lacto-N-tetraose (LNT). **(B)** MS characterization of the key intermediate product LNT-Tag2. ESI-MS *m/z* calculated for C_41_H_60_N_2_O_29_S_2_ [M - 2H]^2−^ 554.52 and found to be 554.14. The peak corresponding to Lac-Tag2 (calculated for C_27_H_37_NO_19_S_2_ [M - 2H]^2−^ 371.86) was not detected. **(C)** MS characterization of the LNT. ESI-MS *m/z* calculated for C_26_H_45_NO_21_Na [M + Na]^+^ 730.62 and found to be 730.23.

The automatic process for enzymatic synthesis of oligosaccharides on the DMF platform was similar to that of degradation of oligosaccharides. Two enzymatic modules were used to convert lactose modified at the reducing end with another sulfonate tag (Lac-Tag2) into Lacto-*N*-tetraose-Tag2 (LNT-Tag2) and, finally, into LNT (Figure 5A). As aforementioned, the droplet containing Lac-Tag2, ATP, and UTP and another one containing enzymatic module 1 (including NahK, GlmU, LgtA, PpA), MgCl_2_, buffer, and GlcNAc were mixed and incubated for 1 h. Then, lacto-*N*-triose II-Tag2 (LNT II-Tag2), the formed product with the sulfonate Tag2, was quantitatively captured onto DEAE magnetic beads, and the rest of the reaction components including enzymes and excess monosaccharides, and NTPs were separated with magnetic beads by a magnet. Then, the captured intermediate was washed in turn with H_2_O and 60 mM NH_4_HCO_3_ and was finally released by washing with 0.3 M NH_4_HCO_3_. Similarly, as the enzymatic synthesis of LNT II-Tag2 by enzymatic module 1, LNT II-Tag2 was further transformed into LNT-Tag2 by the enzymatic module 2 (including GalK, USP, WbgO, PpA). Finally, the Tag2 was removed under mild acidic conditions to give LNT as the final product having a free reducing end. During each step, the intermediates and the final product were characterized by MS. LNT II-Tag2 (calculated for C_35_H_50_N_2_O_24_S_2_ as [M - 2H]^2−^ 473.46 and found to be 473.01), LNT-Tag2 (calculated for C_41_H_60_N_2_O_29_S_2_ as [M - 2H]^2−^ 554.52 and found to be 554.14), and LNT (calculated for C_26_H_45_NO_21_Na as [M + Na]^+^ 730.62 and found to be 730.23) were observed (Figures 5B, C; Supplementary Figure S6). The extent of the reaction in DMF was not measured since it was difficult to quantify by HPLC for the substrates or products in a droplet. Nevertheless, the peak corresponding to Lac-Tag2 (calculated for C_27_H_37_NO_19_S_2_ [M - 2H]^2−^ 371.86) was not detected based on MS. Therefore, it was speculated that nearly all substrates were converted into the product lacto-N-tetraose. These results proved that LNT was successfully synthesized on the DMF platform through an automatic process.

## 4 Conclusion

Enzymatic degradation of tetra-*N*-acetyl chitotetraose (A4) and enzymatic synthesis of lacto-*N*-triose II (LNT II) were achieved successfully on our DMF platform. Moreover, a strategy integrating enzymatic oligosaccharide degradation or synthesis and magnetic manipulation to realize the separation and heterogeneous purification process after enzymatic reactions on the DMF platform was designed. Using this strategy, automatic enzymatic degradation of tetra-*N*-acetyl chitotetraose modified with Tag1 (A4-Tag1) and automatic enzymatic synthesis of lacto-*N*-tetraose (LNT) were realized. In conclusion, we proved the feasibility of enzymatic degradation and synthesis of oligosaccharides and developed an automatic process on the DMF platform by combining with magnetic beads-based separation and purification. Here, the DMF platform exhibits the potential capabilities of versatile, multiplexed, and automatable biochemical operations like oligosaccharide degradation and synthesis. In the future, our DMF platform would be integrated with micropumps for pipelined reactions and instruments for *in situ* detection so that the whole automatic process can be realized under the control of integrated programs. Above all, this work would lay the foundation for the development of automatic enzymatic glycan synthesizers and sequencers based on DMF.

## Data Availability

The original contributions presented in the study are included in the article/Supplementary Material; further inquiries can be directed to the corresponding authors.
