# Supplementary material for: Digital microfluidics-engaged automated enzymatic degradation and synthesis of oligosaccharides
Source: Front Bioeng Biotechnol. 2023 Jun 21;11:1201300. doi: 10.3389/fbioe.2023.1201300 (PMC10320006; doi:10.3389/fbioe.2023.1201300)
Supplement: Supplementary file 1 [file Table1.DOCX]

Supplementary Material

Digital microfluidics-engaged automated enzymatic degradation and synthesis of oligosaccharides

Yunze Sun, ‡^1,2^ Yiran Wu, ‡^1,2^ Dachuan Ma, ^3^ Jian-Jun Li, *^2^ Xianming Liu, ^3^ Yuanjiang You, ^2^ Jun Lu, ^4^ Zhen Liu, ^5^ Xin Cheng ^5^and Yuguang Du *^2^

^1^University of Chinese Academy of Sciences, Beijing 100049, China.

^2^State Key Laboratory of Biochemical Engineering, Institute of Process Engineering, Chinese Academy of Sciences, Beijing 100190, China.

^3^Department of Biotechnology, Dalian Institute of Chemical Physics, Chinese Academy of Sciences, Dalian, Liaoning 116023, China. Emails:

^4^Institute of Physics, Chinese Academy of Sciences, Beijing 100190, China.

^5^Department of Materials Science and Engineering, Southern University of Science and Technology, Shenzhen 518055, China

‡These authors contributed equally to this work.

*** Correspondence:**

Corresponding authors: Jian-Jun Li, E-mail: jjli@ipe.ac.cn; Xianming Liu, E-mail: liuxianming@dicp.ac.cn; Yuguang Du, E-mail: ygdu@ipe.ac.cn.

# Supplementary Data

## Supplementary Figures


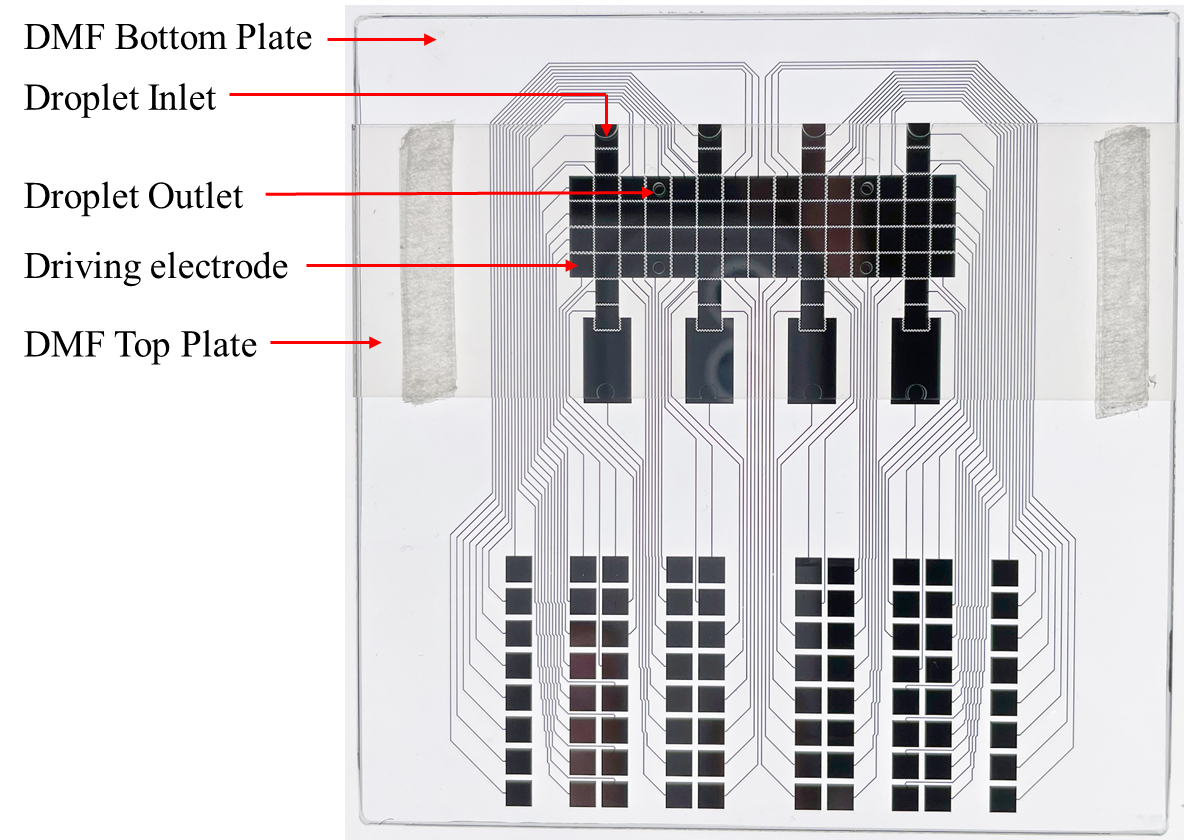


**Supplementary Figure S1.** The physical picture of digital microfluidic device.


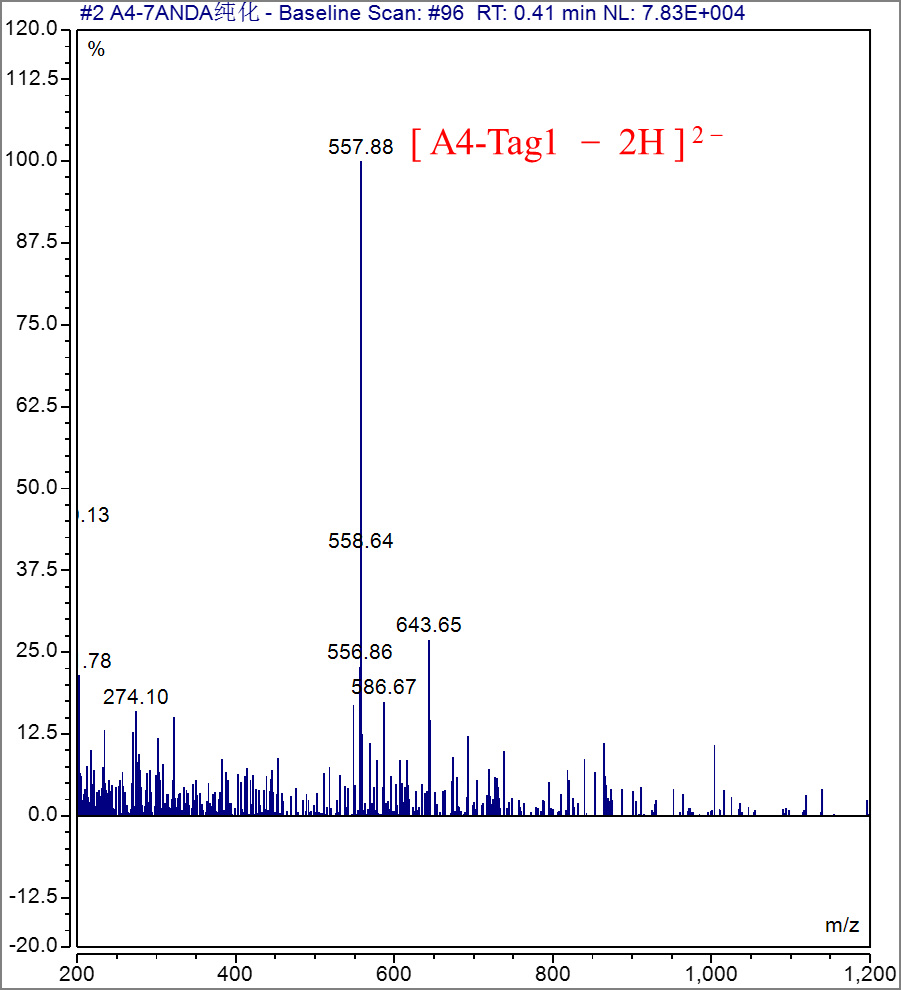


**Supplementary Figure S2.** MS characterization of A4-Tag1. ESI-MS m/z calcd for C_42_H_59_N_5_O_26_S_2_ [M－2H]^2-^ 557.03, found 557.88.


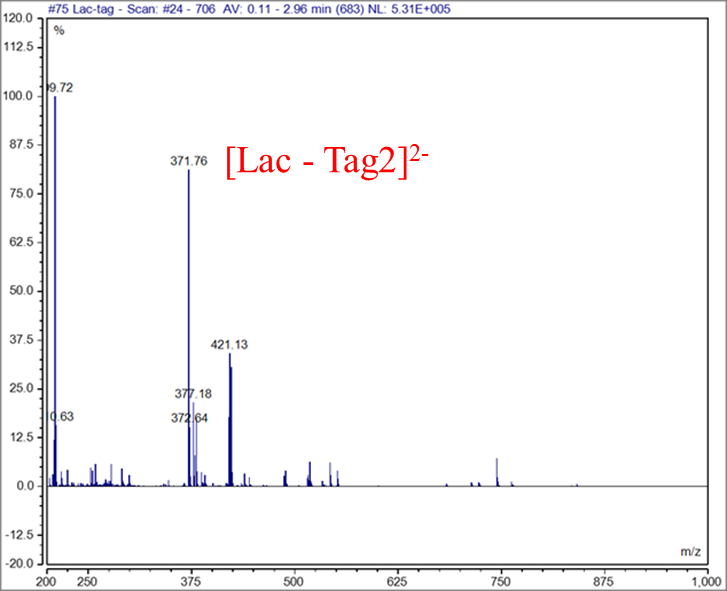


**Supplementary Figure S3.** MS characterization of Lac-Tag2. ESI-MS m/z calcd for C_27_H_37_NO_19_S_2_ [M－2H]^2-^ 371.86, found 371.76.


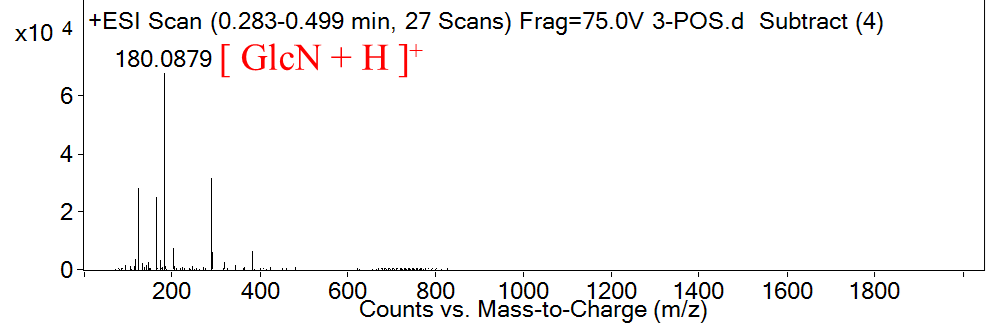


**Supplementary Figure S4.** MS characterization of the degradation product of RX14. ESI-MS m/z calcd for C_6_H_13_NO_5_ [M + H]^+^ 180.21, found 180.0879.


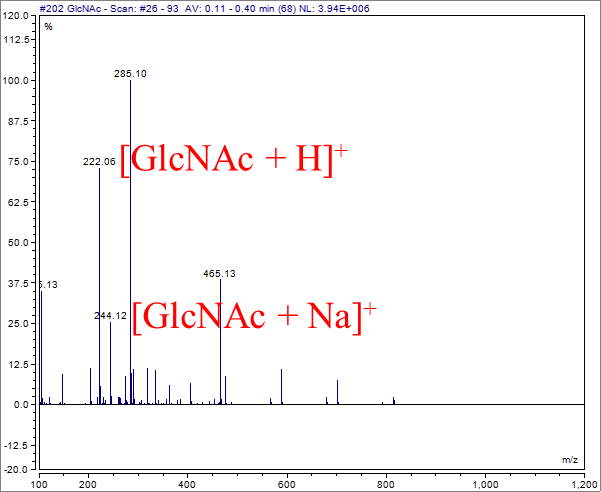


**Supplementary Figure S5.** MS characterization of droplet① in the process of degradation of A4-Tag1. ESI-MS m/z calcd for C_8_H_16_NO_6_ [M + H]^+^ 222.22, found 222.05. ESI-MS m/z calcd for C_8_H_15_NO_6_Na[M + Na]^+^ 244.20, found 244.12.


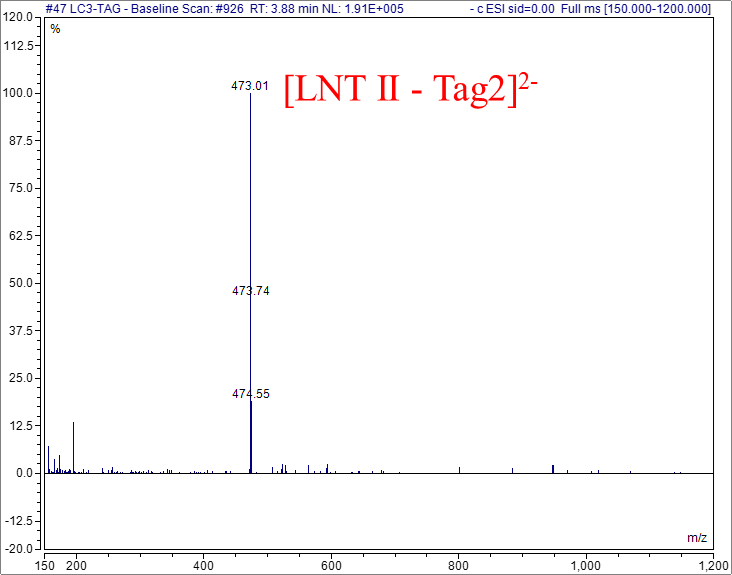


**Supplementary Figure S6.** MS characterization of the product LNT II-Tag2. ESI-MS m/z calcd for C_35_H_50_N_2_O_24_S_2_ [M－2H]^2-^ 473.46, found 473.01.

## Supplementary Tables

**Supplementary Table S1**. Components of enzymatic modules.

|  | Enzyme | Concentration (mg/mL) |
| --- | --- | --- |
| Enzymatic Module 1 | NahK | 0.5 |
|  | PPA | 0.25 |
|  | GlmU | 0.5 |
|  | LgtA | 0.4 |
| Enzymatic Module 2 | GalK | 0.5 |
|  | PPA | 0.25 |
|  | β3GalT | 0.5 |
|  | USP | 0.5 |

## Supplementary Videos

**Supplementary Videos S1.** The process of oligosaccharide degradation on the DMF platform

Enzymatic reaction of oligosaccharide degradation on DMF chip.mp4

**Supplementary Videos S2.** The automatic process of oligosaccharide degradation on the DMF platform

Automatic process of oligosaccharide degradation on DMF chip.mp4
